# Comparison of commercial and in-house Real-time PCR assays for quantification of Epstein-Barr virus (EBV) DNA in plasma

**DOI:** 10.1186/1471-2180-7-22

**Published:** 2007-03-28

**Authors:** Francesca Perandin, Elisabetta Cariani, Caterina Patrizia Pollara, Nino Manca

**Affiliations:** 1 Department of Experimental and Applied Medicine, Section of Microbiology, University of Brescia, P.le Spedali Civili, 25123 Brescia, Italy

## Abstract

**Background:**

Epstein-Barr virus (EBV) DNA load monitoring is known to be useful for the diagnosis and monitoring of EBV-associated diseases. The aim of this study is to compare the performance of two real-time PCR assays for EBV DNA: a commercial kit as the Q-EBV Real-Time System (Q-EBV PCR, Amplimedical, Turin, Italy) and an in-house assay (EBV RQ-PCR).

**Results:**

The range of linearity and the degree of precision of the two assays were similar. The clinical sensitivity of Q-EBV PCR was higher for reference samples containing less than 1,000 EBV DNA copies/ml. The absolute quantitative results of the two methods were statistically correlated (R^2 ^= 0.7789; p < 0.0001), with the systematic overestimation by EBV RQ-PCR possibly linked to different amplification efficiency in calibration standards.

**Conclusion:**

Both the commercial and the in-house assay may be appropriate for clinical use, but common standards are advisable for comparable absolute values, as these would improve the clinical utility of EBV DNA load measurement.

## Background

Epstein-Barr virus (EBV) is a ubiquitous human B-lymphotropic herpesvirus that infects more than 90% of the world's population and establishes a lifelong (usually asymptomatic) infection in its host. It has been estimated that the number of EBV-infected B cells is controlled in healthy individuals by EBV-specific immunity [1]. Nevertheless, EBV is the causative agent of infectious mononucleosis and is associated with several malignant proliferative disorders such as Burkitt's lymphoma, Hodgkin's lymphoma, some B- and T-cell non-Hodgkin's lymphomas, and nasopharyngeal and gastric carcinoma [2-5]. In immunocompromised subjects, active EBV infection is a strong risk factor for the development of post-transplant lymphoproliferative disease (PTLD) and AIDS-related lymphoma [6].

Quantitative molecular assays for the assessment of viral load have helped to describe and monitor EBV-related diseases. Although highly sensitive, however, conventional quantitative PCR is rather laborious and time-consuming. In contrast, real-time amplification technology can overcome these difficulties. A range of different assay formats and protocols involving TaqMan probes [7,8] and fluorescence resonance energy transfer probes [9-12] have been reported and various in-house and commercial assays for EBV load measurement are available [3,7-10,13,14]. However, the considerable differences in EBV load detected by quantitative assays [15] constitute an unsolved problem. This study compares the performance of two real-time PCR assays for EBV DNA: a commercial kit (Q-EBV PCR; Amplimedical, Turin, Italy) and an in-house assay (EBV RQ-PCR) [13].

## Results

**Figure 1:**
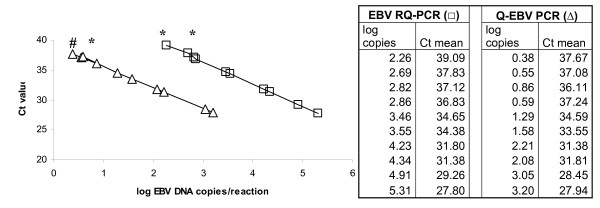
Correlation of EBV DNA load between EBV RQ-PCR (in-house assay: blank squares; R^2^: 0.999) quantification assay and Q-EBV PCR (commercial assay: blank triangles; R^2^: 0.999). The table lists Ct values and log copies. Copies were obtained by both real-time methods. Ct: Cycle threshold # = 1/3 replicates; * = 2/3 replicates.

In order to evaluate the dynamic range of the assays, 10 serial five-fold dilutions of culture supernatant derived from an EBV-positive cell line were tested in triplicate by both methods. These were prepared since high-titre reference material was not available. For each method, linear results were obtained up to 3-log dilution, whereas the three further five-fold dilutions showed comparable quantitative results. Only 1–2 out of 3 replicates were detected in the last two possible dilutions, while 10^-5 ^dilution was negative in both assays. Altogether, the in-house assay (EBV RQ-PCR) showed a remarkable overestimation (on average, about 2 log) of quantitative results, as compared to the commercial assay (Q-EBV PCR) (Figure 1). To establish the level of precision, inter- and intra-assay variability was determined by amplifying replicates of three dilutions of EBV-positive culture supernatant using the same DNA as a sample in both methods (Table 1). Intra-assay variability of the Q-EBV PCR and EBV RQ-PCR methods was determined by amplifying all samples in quadruplicate, whereas inter-assay variability was determined by amplifying the three dilutions in triplicate in four independent experiments. Overall, the last dilution reached in both methods the highest coefficient of variation and linearity lack for values near or just below the cut-off level. Moreover, the in-house EBV RQ-PCR reached a slightly higher intra-assay precision and lower inter-assay precision, compared to the commercial assay.

The accuracy of the two methods was evaluated using a Quality Control for Molecular Diagnosis (QCMD) EBV 2003 proficiency panel. Aliquots of QCMD samples were extracted and aliquots of the DNA samples were analysed at the same time by each assay, avoiding repeated freeze-thaw cycles. Both methods correctly classified the only negative sample (QC2) and all the samples (QC1, QC3, QC5 and QC6) containing more than 1,000 copies/ml, as determined by EM. Only Q-EBV-PCR detected three more samples with a viral load of 100–1,000 copies/ml (QC4, QC7 and QC8), though not all replicates were positive (Table 2). Quantitative results indicated an overestimation by EBV RQ-PCR (mean = 1.28 log) and an underestimation by Q-EBV-PCR (mean = 0.54 log), as compared to EM quantification.

**Figure 2:**
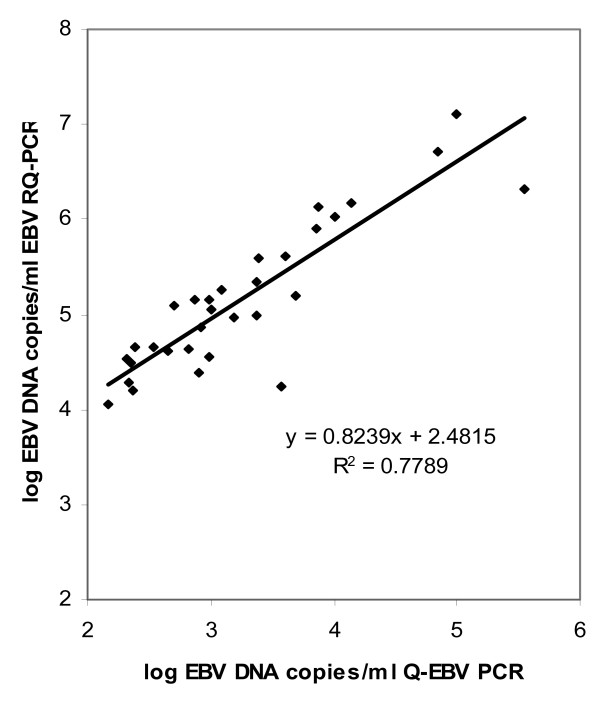
Correlation of EBV DNA loads by EBV RQ-PCR (in-house PCR) and Q-EBV PCR in 31 samples positive in both assays.

**Figure 3:**
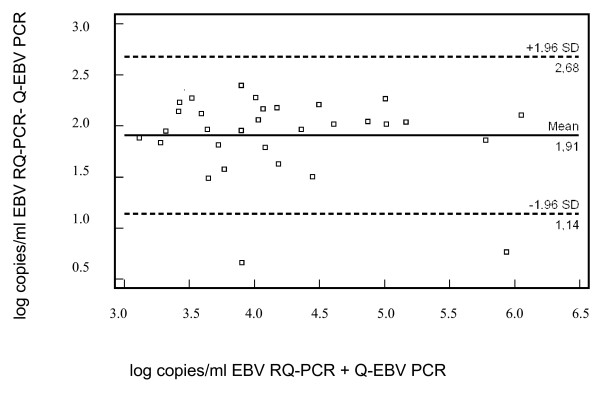
Differences from the mean for all positive samples by EBV RQ-PCR and Q-EBV PCR quantification assays.

Of 199 consecutive plasma samples received by our laboratory for EBV DNA quantification, 17 were positive and 182 negative by both methods. All the samples were monitored for the presence of inhibitors by adding internal positive controls, beta globin and actin DNA, and no negative results or partial amplification were observed. All the 50 plasma samples from healthy blood donors were negative in both assays. The mean copy numbers for the 17 positive plasma samples was respectively 23,583 copies/ml (for Q-EBV-PCR) and 360,256 copies/ml (for EBV RQ-PCR) (p < 0.0001). Considering all the 31 samples positive to both tests and including the QCMD EBV panel samples and serial dilutions of culture supernatant obtained from immortalized clinical specimen lymphocytes, the results of the two methods were statistically correlated (R^2 ^= 0.7789; p < 0.0001) (Figure 2). A systematic difference was noted by plotting the mean value of each positive sample tested by both assays against the difference between the results of the two methods (Figure 3), with about 1.9 log mean overestimation by EBV RQ-PCR.

The different levels of circulating EBV DNA measured by each assay might be due to a difference in PCR efficiency. PCR efficiency was calculated by the equation E = [10^-1/slope of the standard curve^]-1. The mean percentage of PCR efficiency, based on 5 consecutive experiments measuring inter- and intra-assay variability, was 96.92% for Q-EBV PCR and 90.41% for EBV RQ-PCR. One of the variables possibly affecting PCR efficiency is the source of DNA used as a standard for quantification (plasmid DNA for Q-EBV-PCR or genomic DNA for EBV RQ-PCR) [12]. To test this hypothesis, samples of Namalwa cellular DNA used in the standard curve of the in-house method were amplified in the commercial assay: EBV DNA values were on average 17.7 fold lower than expected (range 14–20). By contrast, when Namalwa DNA dilutions were used as a standard in the same experiment, PCR efficiency was about 8% lower (84.6% versus 92.3%). The amplification of the plasmid standards enclosed in the Q-EBV PCR kit yielded copy numbers 14.4 fold higher than the expected average (range 11.3–18).

## Discussion

The quantification of EBV DNA load is useful for detecting viral reactivation, which increases the risk of PTLD in immunosuppressed patients [16,17], and monitoring antiviral therapy. In addition, recent studies suggest that EBV DNA fragments are released from tumour cells in nasopharyngeal carcinoma and EBV-positive lymphomas [18-22]. In such cases, the EBV DNA load may be used as a surrogate disease activity marker with potential applications in clinical monitoring and prognostication. In order to determine the clinical significance of an EBV load, it might be better to monitor viral load dynamics in patients by quantification of EBV load in peripheral mononuclear cells (PBMCs), though EBV DNA is detectable during active infection for a longer period in whole blood than in plasma [23,24]. However, the real-time PCR protocol used in these assays is preferable for the quantification of viral load in plasma or liquor.

Today a wide variety of quantitative PCR assays are available for EBV DNA quantification, including quantitative-competitive PCR and real-time PCR (TaqMan PCR and LightCycler PCR) [3,8-10,12-14]. They are faster than conventional quantitative PCR and employ a close-tube system that eliminates the need for post-PCR analysis, thus reducing the risk of contamination. Although real-time PCR assays are known to be of excellent diagnostic value in certain clinical settings, interlaboratory standardization of EBV DNA load monitoring has yet to be achieved [15].

We compared the performance of two real-time PCR assays for EBV DNA quantification in plasma, one of which is commercial (Q-EBV PCR) and the other in-house (EBV RQ-PCR). False negative results, due to the presence of inhibitors, did not occur and none of the samples inhibited amplification of the internal positive control in either of the real-time PCR assays. These results confirm that the method used for DNA extraction does provide an inhibitor-free target for quantitative assessment by PCR.

Several factors have been linked to variation in PCR results, including PCR reagents, primer stability and specificity, and PCR product size [25]. The choice of target region for amplification is known to influence significantly the results of quantitative EBV DNA assays. One of the most widely used PCR targets, the BamHI-W region, occurs in multiple repeats which can vary considerably across naturally occurring EBV isolates. For this reason, the EBV DNA load can be up to 5-fold higher when BamHI-W primers are used, as compared to primers located in single-copy EBV genes [22]. This observation, however, does not account for the discrepancies observed in the present study, as both assays amplify the single-copy EBNA-1 gene.

Real-time PCR quantification relies on the assumption that amplification efficiencies are the same for patient samples and in the standard used for calibration. Low amplification efficiency in patient samples, compared to the calibrator, lead to underestimation of the DNA content in samples [26]. Alternatively, it can be argued that the decreased PCR efficiency of standards may lead to overestimation of DNA samples. The DNA standard source used to build a calibration curve appears to be crucial in determining the final outcome of quantification [19,22]. Indeed, we observed a lower rate of efficiency in the in-house assay, using a genomic standard, as compared to the commercial assay, which employs a plasmid standard. The influence of calibrator DNA on the assay's quantitative results is supported by the observation that when genomic DNA was used as a standard in the commercial assay, amplification efficiency was lower, and higher EBV DNA values were measured on plasmid samples containing known amounts of EBV DNA.

## Conclusion

Our results suggest that commercial and in-house assays for the quantification of EBV DNA are both appropriate for clinical use because they perform similarly in analytical terms and generate correlated quantitative results. The use of common standards would help to achieve comparable absolute values, thus limiting the high degree of variability in EBV DNA load shown by various assays [12,27] and improving the diagnostic significance of real-time EBV copy number quantification.

Given the high coefficients of variation involved, it would also help to specify the confidence interval in reported results (as an estimate of uncertainty), so that viral load fluctuations are not misinterpreted as clinically significant data.

## Methods

### Samples

This study included a total of 199 consecutive plasma samples received by the Microbiology Laboratory of Brescia Hospital (Spedali Civili) between June and December 2005 for EBV viral load determination by real-time PCR.

The sensitivity, linear range and precision of the real-time assays were compared using DNA extracted from five-fold dilutions of EBV-positive culture supernatant (B-95.8 cell line). As negative controls for EBV RQ-PCR, we tested 6 supernatants of viral cultures from Herpes Simplex Virus type 1 (n = 4) and type 2 (n = 2) seropositive patients, who were also positive by PCR hybridized on Gen-ETI-K™ DEIA (DiaSorin, Vercelli, Italy), and 50 plasma samples from healthy blood donors. The B-95.8 EBV-positive cell line and 6 EBV positive cell lines, immortalized in our laboratory from patients with Mycosis Fungoides [28] served as positive controls for EBV-specific amplification.

In order to assess the accuracy of molecular amplification, samples from the Quality Control for Molecular Diagnostic (QCMD) 2003 Epstein-Barr virus proficiency panel were analyzed in triplicate. The panel consisted of 7 frozen plasma samples containing different concentrations of electron microscope (EM)-quantified EBV samples and one EBV-negative plasma sample.

### B-95.8 cell line

The marmoset blood leukocytes B-95.8 EBV transformed cell line is a continuous line that releases high titres of transforming EBV (ATCC CRL 1612). The DNA was extracted from the culture supernatant using QIAamp DNA Mini (Qiagen, Hilden, Germany) as reported by the manufacturer and the DNA serial dilution was made with sterile water.

### Commercial assay (Q-EBV PCR)

DNA was extracted by QIAamp DNA Mini (Qiagen, Hilden, Germany) using the "Blood and body fluid protocol" as recommended in the manufacturer's instructions, starting from 200 μl of plasma. It was eluted in a final volume of 50 ul. Q-EBV PCR for a highly conserved region of gene EBNA-1 was performed in 25 μl on 5 μl DNA, according to the manufacturer's instructions, using Q-EBV AmpliMASTER, Q-EBV Amplimix containing the EBNA-1 primer set and Q-EBV AmpliPROBE (Amplimedical). EBV DNA was quantified by Q-EBV AmpliSTANDARD, consisting of serial dilutions of a plasmid containing the target amplification region as multiple tubes of prediluted plasmid, ranging in concentration from 1.0 × 10^5 ^to 1.0 × 10^2 ^copies/reaction, as determined by the manufacturer spectrophotometric reading. As reported in the manufacturer's package insert, the lower detection limit is 10 copies/ml. The reactions were carried out on an ABI PRISM 7300 Analytical PCR System (Applied Biosystems, Foster City, CA), according to the manufacturer's instructions. The PCR cycle protocol consist of 2 min at 50°C, 10 min at 95°C, and 45 two-step cycles of 15 sec each at 95°C and of 1 min at 60°C. This method allows linear quantification of 10^1 ^to 10^6 ^DNA copies per reaction, as stated by the manufacturer. To establish the concentration of EBV DNA copies per ml, the readings were multiplied by 62.5, assuming an 80% mean efficiency of DNA extraction, as suggested by the manufacturer.

Appropriate positive and negative controls were included in all the experiments. As a control for PCR inhibitors and amplification quality, β-globin DNA (CPE-DNA, Amplimedical) was added to each sample before the extraction step, according to the manufacturer's instructions, and amplified with β-globin primers in the same reaction mix as EBV in multiplex PCR.

### In-house assay (EBV RQ-PCR)

DNA was extracted as reported above. The real-time assay was performed as described by Wagner et al. [2001], with minor adjustments. The BAM HI-K region, encoding for EBNA-1 gene, was chosen as an amplification target [13]. Briefly, 5 μl of DNA were amplified with TaqMan Universal Master Mix (Applied Biosystems, Foster City, CA), adjusted to a volume of 25 μl with 50 nM of forward primer (5'-CCGGTGTGTTCGTATATGGAG-3'), 300 nM of reverse primer (5'-GGGAGACGACTCAATGGTGTA-3'), 200 nM of probe (5'VIC-TGCCCTTGCTATTCCACAATGTCGTCTT-TAMRA3') and distilled water. Primers and probe were synthesized by MWG Biotech S.r.l. (Ebersberg, Germany). Each reaction consisted of 2 min at 50°C, 10 min at 95°C, and 40 two-step cycles of 15 sec each at 95°C and of 1 min at 60°C. For quantification of EBV DNA, serial 10-fold dilutions of DNA extracted from Namalwa cells were used as a standard, ranging in concentration from 1.0 × 10^5 ^to 1.0 × 10^2 ^copies/reaction, as quantified by spectrophotometry, diluted in sterile water and used directly in PCR. Since Namalwa cells contain two integrated EBV copies per cellular genome, it was possible to calculate the number of target EBV genomes from the cellular Namalwa DNA amount using the equation: 33 pg of Namalwa DNA contain 10 EBV genome copies. Each reaction was performed using an ABI PRISM 7700 analytical PCR system (SDS version 1.7) (Applied Biosystems) with the amplification and detection conditions set by the instrument. The detection limit of the assay is 3.3 pg of Namalwa DNA, equivalent to 1 EBV genome per reaction [13]. The EBV DNA load per ml was calculated as described for the Q-EBV PCR assay. Positive and negative controls were included in all the experiments and each sample was run in duplicate for each PCR. As a control for PCR inhibitors, β-actin amplification (TaqMan Beta-Actin Detection Reagents, Applied Biosystems) was performed in multiplex with EBV, according to the manufacturer's guidelines.

### Statistics

The differences in viral load values were calculated using a Wilcoxon W test for paired samples. Intra- and inter-assay variations were evaluated by descriptive statistics. The relationship between viral loads was assessed by regression analysis. Bland and Altman's method was adopted to analyze the agreement between viral load measurements of the positive samples. Statistical data was processed using MedCalc 8.1.1.0 software, with a p value < 0.05 judged to be statistically significant.

## Authors' contributions

PF designed the study, and drafted the manuscript with EC; PCP contributed to data collection; MN revised the manuscript. All the authors read and accepted the final version of this paper.

## Figures and Tables

**Table 1:** Intra- and inter-assay reproducibility of EBV RQ-PCR in-house assay and Q-EBV PCR quantification kit tested on serial dilutions of culture supernatant.

**Comparison**	**Method**	**Dilution**	**Mean EBV DNA load (copies/reaction)**	**ST. DEV.**	**CV (%)**
INTRA-ASSAY	EBV RQ-PCR	Und.	19511	2557	13
		1:10	5403	420	8
		1:100	2055	605	29
	Q-EBV PCR	Und.	1852	312	17
		1:10	75	12	16
		1:100	12	4	36
INTER-ASSAY	EBV RQ-PCR	Und.	43774	16467	28
		1:10	2406	544	35
		1:100	1116	618	37
	Q-EBV PCR	Und.	4363	998	21
		1:10	241	84	24
		1:100	22	7	31

ST. DEV.: Standard Deviation; CV: Coefficient of Variation; Und.: undiluted.

**Table 2:** QCMD EBV 2003 panel results by EBV RQ-PCR and Q-EBV PCR quantification assay.

QCMD sample	Q-EBV-PCR		EBV RQ-PCR	
	(copies/ml)	(Ct)	(copies/ml)	(Ct)

EM copies/ml				

QC1-10000	2350	33.26	99424	35.35
QC2-1	1	40.00	1	40.00
QC3-2500	220	37.01	19548	37.90
QC4-500	265	36.90 §	1	40.00
QC5-5000	1535	33.89	94985	35.48
QC6-1000	205	36.85 §§	34737	37.36
QC7-100	81.25	38.20 §	1	40.00
QC8-250	134.4	37.46 §	1	39.50

EM: electron microscope; Ct: Cycle threshold

§= 1/3 replicates; §§ = 2/3 replicates

## References

[B1] Wagner HJ, Bein G, Bitsch A, Kirchner H (1992). Detection and quantification of latently infected B lymphocytes in Epstein-Barr virus-seropositive, healthy individuals by polymerase chain reaction. J Clin Microbiol.

[B2] Young LS, Murray PG (2003). Epstein-Barr virus and oncogenesis: from latent genes to tumors. Oncogene.

[B3] Timms JM, Bell A, Flavell JR, Murray PG, Rickinson AB, Traverse-Glehen A, Berger F, Delecluse HJ (2003). Target cells of Epstein-Barr-virus (EBV)-positive post-transplant lymphoproliferative disease: similarities to EBV-positive Hodgkin's lymphoma. Lancet.

[B4] zur Hausen H, Schulte-Holthausen H, Klein G, Henle W, Henle G, Clifford P, Santesson L (1970). EBV DNA in biopsies of Burkitt tumors and anaplastic carcinomas of the nasopharynx. Nature.

[B5] Osato T, Imai S (1996). Epstein-Barr virus and gastric carcinoma. Seminar Cancer Biol.

[B6] Rowe DT, Webber S, Schauer EM, Reyes J, Green M (2001). Epstein-Barr virus load monitoring: its role in the prevention and management of post-transplant lymphoproliferative disease. Transplant Infect Dis.

[B7] Kimura H, Nishikawa K, Hoshino Y, Sofue A, Nishiyama Y, Morishima T (1999). Quantitative analysis of Epstein-Barr virus load by using a real-time PCR assay. J Clin Microbiol.

[B8] Dehee A, Asselot C, Piolot T, Jacomet C, Rozenbaum W, Vidaud M, Garbarg-Chenon A, Nicolas JC (2001). Quantification of Epstein-Barr virus load in peripheral blood of human immunodeficiency virus-infected patients using real-time PCR. J Med Virol.

[B9] Brengel-Pesce K, Morand P, Schmuc A, Bourgeat MJ, Buisson M, Bargues G, Bouzid M, Seigneurin JM (2002). Routine use of real-time quantitative PCR for laboratory diagnosis of Epstein-Barr virus infection. J Med Virol.

[B10] Patel S, Zuckerman M, Smith M (2003). Real-time quantitative PCR of Epstein-Barr virus BZLF1 DNA using the LightCycler. J Virol Methods.

[B11] Ruiz G, Pilar P, De Ory F, Echevarrìa JE (2005). Comparison of commercial real-time PCR assays for quantification of Epstein-Barr virus DNA. J Clin Microbiol.

[B12] Krumbholz A, Meerbach A, Zell R, Gruhn B, Henke A, Birch-Hirschfeld E, Wutzler P (2006). Comparison of a LightCycler-based real-time PCR for quantitation of Epstein-Barr viral load in different clinical specimens with semiquantitative PCR. J Med Virol.

[B13] Wagner HJ, Wessel M, Jabs W, Smets F, Fischer L, Offner G, Bucsky P (2001). Patients at risk for development of posttransplant lymphoproliferative disorder: plasma versus peripheral blood mononuclear cells as material for quantification of Epstein-Barr viral load by using real-time quantitative polymerase chain reaction. Transplantation.

[B14] Niester HG, van Esser J, Fries E, Wolthers KC, Cornelissen J, Osterhaus AD (2000). Development of a real-time quantitative assay for detection of Epstein-Barr virus. J Clin Microbiol.

[B15] Stevens SJ, Verschuuren EA, Pronk I, van Der Bij W, Harmsen MC, The TH, Meijer CJ, van Den Brule AJ, Middeldorp JM (2001). Frequent monitoring of Epstein-Barr virus DNA load in unfractionated whole blood is essential for early detection of posttransplant lymphoproliferative disease in high-risk patients. Blood.

[B16] van Esser JW, van der Holt JB, Meijer E, Niester HGM, Trenschel R, Thijsen SFT (2001). Epstein-Barr virus reactivation is a frequent event after allogenic hematopoietic stem cell transplantation and quantitatively predicts EBV-lymphoproliferative disease following T-cell depleted stem cell transplantation. Blood.

[B17] Gandhi MK, Lambley E, Burrows J, Dua U, Elliot S, Shaw PJ, Prince MH, Wolf M, Clarke K, Underhill C, Mills T, Mollee P, Gill D, Mollee P, Gill D, Marlton P, Seymour JF, Khanna R (2006). Plasma Epstein-Barr virus (EBV) DNA is a biomarker for EBV-positive Hodgkin's lymphoma. Clin Cancer Res.

[B18] Au W-Y, Pang A, Choy C, Chim C-S, Kwong Y-L (2004). Quantification of circulating Epstein-Barr virus (EBV) DNA in the diagnosis and monitoring of natural killer cell and EBV-positive lymphomas in immunocompetent patients. Blood.

[B19] Chan KCA, Zhang J, Chan ATC, Lei KIK, Leung S-F, Chan LYS, Chow KCK, Lo YMD (2003). Molecular characterization of circulating EBV DNA in the plasma of nasopharyngeal carcinoma and lymphoma patients. Cancer Res.

[B20] Lo YMD, Chan AT, Chan LY, Leung SF, Lam CW, Huang DP, Johnson PJ (2000). Molecular prognostication of nasopharyngeal carcinoma by quantitative analysis of circulating Epstein-Barr virus DNA. Cancer Res.

[B21] Le T, Jones CD, Yau T-K, Shirazi HA, Wong PH, Thomas N, Patterson BK, Lee AWM, Zehnder JL (2005). Comparison study of different PCR assays in measuring circulating plasma Epstein-Barr virus DNA levels in patients with nasopharyngeal carcinoma. Cancer Res.

[B22] Wong ML, Medrano JF (2005). Real-time PCR for mRNA quantitation. BioTechniques.

[B23] Fafi-Kremer S, Bregel-Pesce K, Bargues G, Bourgeat MJ, Seigneurin JM, Morand P (2004). Assessment of automated DNA extraction coupled with real-time PCR for measuring Epstein-Barr virus load in whole blood, peripheral mononuclear cells and plasma. J Clin Virol.

[B24] Kozic S, Vince A, B es JI, Rode OD, Lepej SZ, Poljak M, Bozic M, Kessler HH (2006). Evaluation of commercial real-time PCR assay for quantification of Epstein-Barr virus DNA in different groups of patients. J Virol Meth.

[B25] Meijerink J, Mandigers C, Van de Locht L, Tonnissen E, Goodsaid F, Raemaekers J (2001). A novel method to compensate for different amplification efficiencies between patient DNA samples in quantitative real-time PCR. J M D.

[B26] Niester HGM (2004). Summary results QCMD 2003 Epstein-Barr virus proficiecy programme.

[B27] Wandinger KP, Jabs W, Siekhaus A, Bubel S, Trillenberg P, Wagner HJ, Wessel K, Kirchner H, Hennig H (2000). Association between clinical disease activity and Epstein-Barr virus reactivation in MS. Neurology.

[B28] Manca N, Piacentini E, Gelmi M, Calzavara P, Manganoni MA, Glukhov A, Gargiulo F, de Francesco M, Pirali F, de Panfilis G, Turano A (1994). Persistence of human T cell lymphotropic virus type 1 (HTLV-1) sequences in peripheral blood mononuclear cells from patients with mycosis fungoides. J Exp Med.

